# In Vitro Effects of Boric Acid on Cell Cycle, Apoptosis, and miRNAs in Medullary Thyroid Cancer Cells

**DOI:** 10.1007/s12011-024-04188-3

**Published:** 2024-04-30

**Authors:** Onurcan Yıldırım, Mücahit Seçme, Yavuz Dodurga, Gülçin Abban Mete, Semin Melahat Fenkci

**Affiliations:** 1 Department of Internal Medicine, Ege University School of Medicine, Izmir, 35100 Turkey; 2 Department of Medical Biology, Ordu University School of Medicine, Ordu, Turkey; 3 Department of Medical Biology, Pamukkale University School of Medicine, Denizli, Turkey; 4 Department of Histology and Embriology, Pamukkale University School of Medicine, Denizli, Turkey; 5 Department of Endocrinology and Metabolism, Pamukkale University School of Medicine, Denizli, Turkey

**Keywords:** Medullary thyroid cancer, Boric acid, TT cells, Apoptosis, miRNA, Cell cycle

## Abstract

Medullary thyroid cancer (MTC) is a highly aggressive and chemotherapy-resistant cancer originating from the thyroid’s parafollicular C cells. Due to its resistance to conventional treatments, alternative therapies such as boric acid have been explored. Boric acid, a boron-based compound, has shown anticarcinogenic effects, positioning it as a potential treatment option for MTC. TT medullary thyroid carcinoma cell line (TT cells) and human thyroid fibroblast (HThF cells) were utilized for the cell culture experiments. Cell viability was assessed using the 2,3-bis(2-methoxy-4-nitro-5-sulfophenyl)-2H-tetrazolium-5-carboxanilide (XTT) assay. Total RNA was extracted using Trizol reagent for gene expression and microRNA (miRNA) analysis via reverse transcription-polymerase chain reaction (RT-PCR). The extent of apoptosis induced by boric acid was determined using the terminal deoxynucleotidyl transferase dUTP nick end labeling (TUNEL) assay. Colony formation assays were conducted to evaluate the impact of boric acid on the colony-forming ability of MTC cells. At 48 h, 50% inhibitory concentration (IC50) of boric acid was found to be 35 μM. Treatment with boric acid resulted in significant modulation of apoptosis-related genes and miRNAs, including increased expression of *phorbol-12-myristate-13-acetate-induced protein 1*(*NOXA), apoptotic protease activating factor 1* (*APAF-1*), *Bcl-2-associated X protein* (*Bax), caspase-3,* and *caspase-9*. In contrast, the expression of *B cell lymphoma 2* (*Bcl2*), *B cell lymphoma‐ extra-large* (*Bcl-xl*), and *microRNA-21 (miR-21)*, which are linked to the aggressiveness of MTC, was significantly reduced. The TUNEL assay indicated a 14% apoptosis rate, and there was a 67.9% reduction in colony formation, as shown by the colony formation assay. Our study suggests that boric acid may have anticancer activity in MTC by modulating apoptotic pathways. These findings suggest that boric acid could be a potential therapeutic agent for MTC and possibly for other malignancies with similar pathogenic mechanisms.

## Introduction

Medullary thyroid cancer (MTC) accounts for 5–10% of all thyroid carcinomas and originates from parafollicular C cells, which are derived from the neural crest and responsible for producing calcitonin [1]. The activation of the RET proto-oncogene is primarily linked to the development of MTC. MTC can be classified into two types: sporadic (sMTC) and hereditary (hMTC). sMTC accounts for approximately 75% of MTC occurrences, while the remaining 25% are hMTC cases, which are often associated with mutations in the RET proto-oncogene [2]. Genetic screening for hMTC can lead to early, potential curative intervention through prophylactic total thyroidectomy. In contrast, sMTC is often only identified after it has metastasized to lymph nodes and distant sites such as bones, liver, and lungs. Total thyroidectomy coupled with lymph node dissection remains the cornerstone of treatment for both forms of MTC [3]. However, systemic chemotherapy typically shows suboptimal response rates and considerable adverse effects. Additionally, the management of advanced metastatic MTC with tyrosine kinase inhibitors has shown limited success [4].

Although RET mutations provide insight into the pathophysiology of MTC, their role, particularly in metastasis, is not fully understood [5]. Recent research has increasingly focused on microRNAs (miRNAs) as critical regulators of gene expression involved in developmental and pathological processes in cancers. MicroRNAs, small non-coding RNAs that regulate gene expression by targeting mRNAs and triggering translational repression or RNA degradation, influence key pathways in cancer progression including cell cycle control and apoptosis. They can act as oncogenes or tumor suppressors, thereby playing a dual role in cancer biology [6]. Understanding specific miRNAs involved in MTC offers significant insights into the tumor’s behavior and potential responsiveness to treatments. Dysregulation of certain miRNAs has been linked to MTC development and progression, impacting processes such as cell proliferation, metastasis, and resistance to apoptosis [3, 7].

Boron, an element typically found in various compounds such as borax, boric acid, colemanite, kernite, ulexite, and borates, is not found in its elemental state in nature. Humans primarily ingest boron in the form of boric acid [8]. Previous research, including both *in vivo* and *in vitro* studies, has highlighted boron’s potential as an anticancer agent and demonstrated efficacy against various types of cancer such as prostate [9, 10], colon [11], lung [12], breast [13], and malignant melanoma [14].

The objective of this study was to investigate the anticancer effects of boric acid on MTC using in vitro models. This investigation specifically focused on how boric acid influences apoptosis and cell proliferation, as well as viability, by modulating gene expression and interacting with key microRNAs associated with the pathogenesis of MTC.

## Material and Methods

### Cell Culture

TT cells (ATCC, CRL 1803TM) and HThF cells (ScienCell Cat No: 3730) were utilized in this study. Both cell lines were cultured at 37 °C in a 5% CO_2_ atmosphere. The growth medium used was Dulbecco’s modified Eagle medium (DMEM; Sigma), supplemented with 10% heat-inactivated fetal bovine serum (FBS; Capricorn Scientific), 20 units/mL penicillin, 20 μg/mL streptomycin, 0.1 mM amino acid solution (Biological Industries), and 1 mM sodium pyruvate (Biological Industries). Boric acid (Eti maden) was applied to the cells in varying concentrations (10 μM, 20 μM, 35 μM, 50 μM, 75 μM, 100 μM, 200 μM, 500 μM) in a time- and dose-dependent manner.

### Cell Proliferation XTT Assay

The effects of boric acid on cell proliferation were assessed using the XTT assay, following the manufacturer’s protocol (Cell Proliferation Assay with XTT Reagent; Biotium Cat No: 30007). TT and HThF cells were seeded into 96-well plates at a density of 1 × 10^4^ cells per well. After 24 h, cells were treated with varying concentrations of boric acid for 24, 48, and 72 h. The dose range was selected based on literature references [9, 15]. Untreated cells served as controls. Post-treatment, the XTT mixture was added, and formazan formation was quantified spectrophotometrically at 450 nm (reference wavelength 630 nm) using a microplate reader (Biotek). Cell viability was calculated as follows:

$$\mathrm{Viability\;}(\mathrm{\%}) =\mathrm{ Absorbance\;of\;experiment\;well}/\mathrm{Absorbance\;of\;control\;well }\times 100$$

IC50 doses were determined using the GraphPad Prism 8 and were employed in subsequent assays such as invasion, migration, TUNEL, real-time PCR, and comet assays.

### RNA Isolation, cDNA Synthesis, and Real-Time PCR (RT-PCR)

Total RNA was isolated from control and treated TT cells using the Trizol Reagent (Invitrogen, USA), following the manufacturer’s guidelines. Complementary DNA (cDNA) was synthesized using the high-capacity cDNA reverse transcription kit (Applied Biosystems, USA).

Gene expression profiles for *caspase-3*, *caspase-9*, *Bcl-2*, *Bcl-xl*, *APAF-1*, *Bax*, and *NOXA* were analyzed using beta-actin as the reference gene. RT-PCR was performed using gene-specific primers, as detailed in Table 1.

**Table 1:** The sequences of primers of genes

*Gene names*	*Primary sequence*
BAX	F: AGAGGATGATTGCCGCCGT R: CAACCACCCTGGTCTTGGATC
Caspase-3	F: GCAGCAAACCTCAGGGAAAC R: TGTCGGCATACTGTTTCAGCA
Bcl-2	F: TTGGCCCCCGTTGCTT R: CGGTTATCGTACCCCGTTCTC
Bcl-xl	F: GGTCGCATTGTGGCCTTTTTC R: AGCTCGGTACCACAGGGTCA
Caspase-9	F: GGCTGTCTACGGCACAGATGGA R: CTGGCTCGGGGTTACTGCCAG
APAF1	F: ATGAGCCCACTCAACAGCAA R: CGCCACCACGCTCTTCTG
NOXA	F: ACCAAGCCGGATTTGCGATT R: ACTTGCACTTGTTCCTCGTGG

*BAX* Bcl-2 associated X, *Bcl-2* B cell lymphoma 2, *Bcl-xl* B cell lymphoma-extra-large, *NOXA* phorbol-12-myristate-13-acetate-induced protein 1, *APAF1* apoptotic peptidase activating factor 1, *F* forward, *R* reverse

Changes in miRNA expression were also assessed using RT-PCR. The miRNA cDNA synthesis kit (Applied Biological Materials Inc.) was used for cDNA synthesis, and relative quantification of *miR-21-5p* and *miR-224-5p* was performed according to the EvaGreen (Applied Biological Materials Inc) Master Mix protocol. miRNA expression was normalized to U6 as the endogenous control.

### TUNEL Assay

The apoptotic impact of boric acid on TT cells was evaluated using the TUNEL assay. Cells from both the control and treatment groups were fixed using 4% (w/v) paraformaldehyde. Apoptosis was subsequently assessed using a commercial TUNEL In Situ Cell Detection Kit (AAT Bioquest), following the manufacturer’s guidelines. The cells were then stained with Hoechst dye and visualized under a fluorescence microscope (Olympus Inc., Tokyo, Japan). In each sample, cells were counted in ten randomly selected fields using the fluorescence microscope. The results were quantified as the percentage of TUNEL-positive cells, which represents the ratio of apoptotic cells to the total number of cells observed.

### Colony Formation Assay

The colony-forming ability of TT cells treated with boric acid was assessed using a colony formation assay. Cells in the exponential growth phase were harvested using trypsin digestion and subsequently counted via the trypan blue dye exclusion method. These cells were then resuspended in DMEM medium supplemented with 10% fetal bovine serum. The resuspended cells were seeded into six-well plates at a density of 1000 cells per well. The culture medium was refreshed every 3 days over a period of 2 to 3 weeks. Once visible colonies had formed in the culture dish, they were fixed with methanol for 10 min and stained using crystal violet. The morphology and number of colonies were then examined and counted under a microscope (Olympus Inc., Tokyo, Japan).

### Study Design

This study outlines two primary experimental conditions for evaluation: a control group consisting of untreated (control) TT cells and a treatment group in which TT cells were exposed to boric acid at the IC50 dose of 35 µM for 48 hours. All assays, except for the XTT assay, were conducted on these two groups. XTT assay was conducted on TT cells and HThF cells with boric acid exposure time- and dose-dependent.

### Statistical Analysis

RT-PCR data were analyzed using the 2^−ΔΔCt^ method and quantified through specialized software. Group comparisons were carried out using “volcano plot” analysis as part of the “RT2 Profiles PCR Array Data Analysis” suite. Statistical assessments were performed using the Student’s *t*-test. For both parametric and non-parametric analyses of the treatment and control groups, IBM SPSS Statistics V21 (IBM Corp., Armonk, NY, USA) was employed. *p*-value of less than 0.05 was considered to be statistically significant. Cells were seeded in separate wells for each experimental condition to maintain the independence of observations. The XTT assay was conducted in six independent experiments, which has been explicitly stated to clarify the sample size and statistical power. Statistical analysis of cell viability was performed using one-way ANOVA and Tukey’s post hoc test to identify significant dose- and time-dependent responses to boric acid treatment. For apoptotic cell proportions and colony formation, the chi-square test was used for statistical analysis.

## Results

### Anti-proliferative Effects of Boric Acid in TT Medullary Thyroid Cancer

**Fig. 1:**
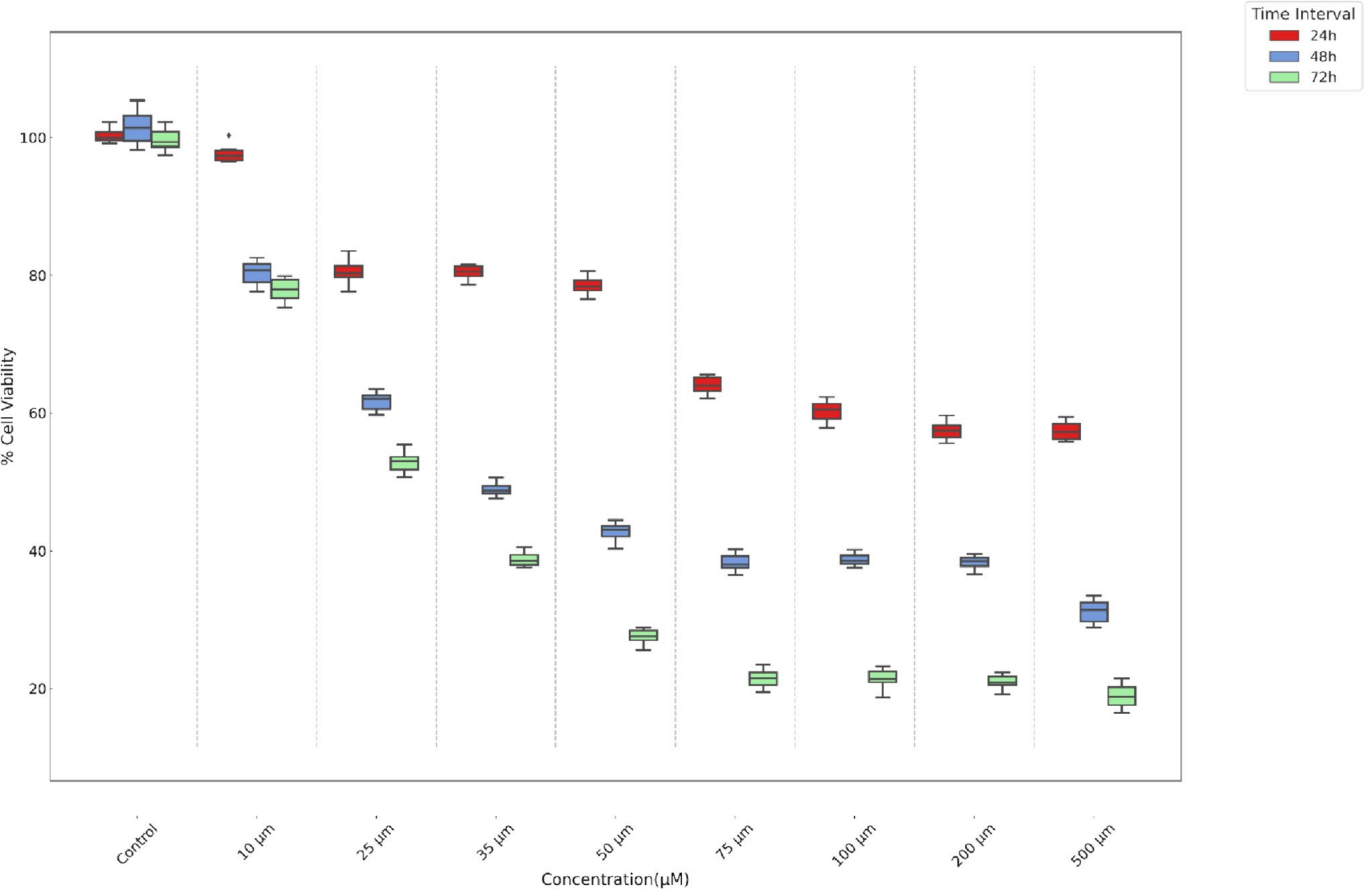
Effect of boric acid on TT cell viability over time and dose. Significant dose effects were observed at all time points (24 h = *p* < 0.05, 48 h = *p* < 0.01, 72 h = *p* < 0.001). Notably, 35 µM boric acid significantly reduced viability at 48 h, marking the IC50 dose (mean concentration is %48.9)

**Fig. 2:**
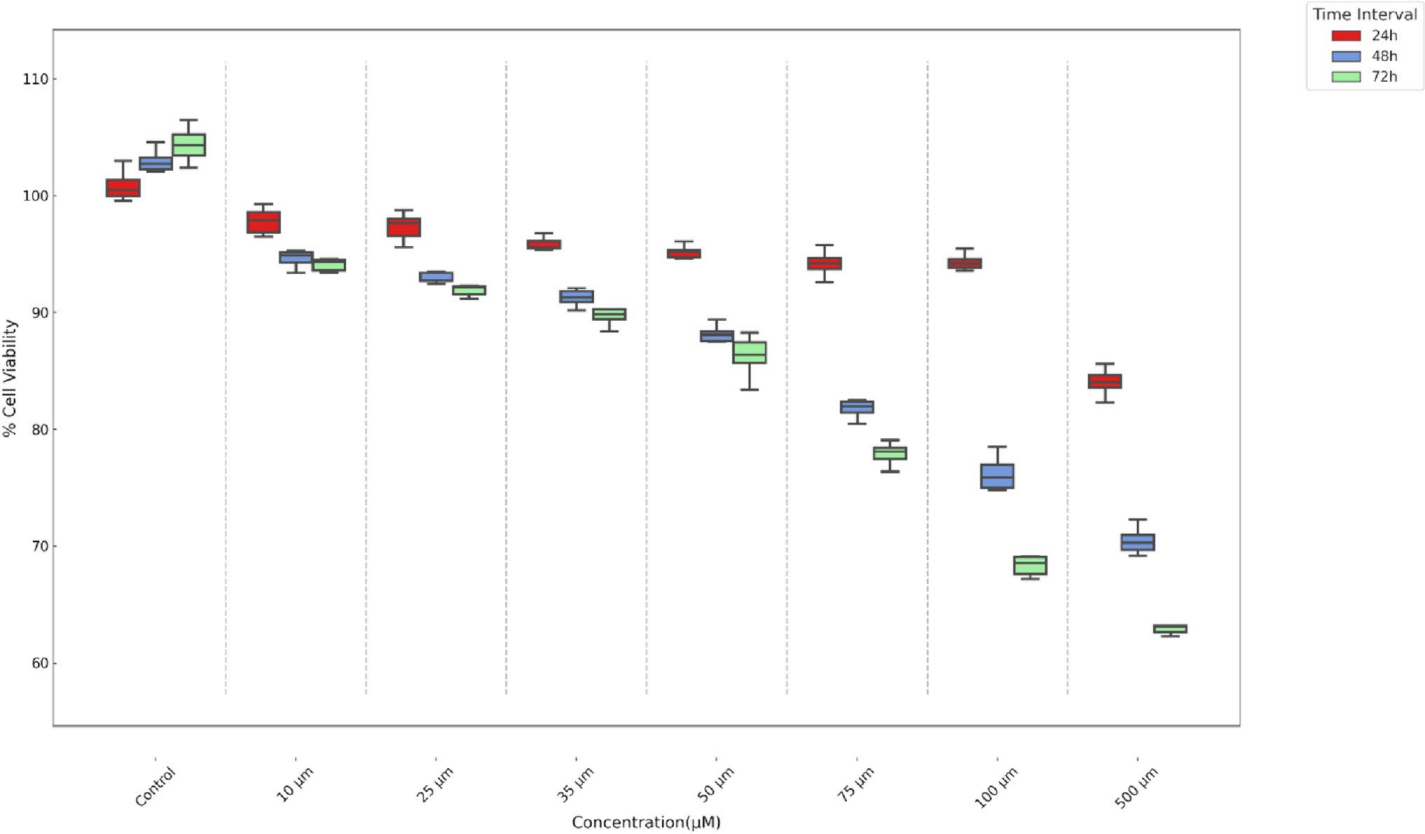
Effect of boric acid on HThF cell viability over time and dose. Significant dose- and time-dependent cytotoxic effects were observed on cell viability (*p* < 0.001 for all time points). Notably, cell viability did not decrease below 50% in any of the tested concentrations or time points

The impact of boric acid on TT cell proliferation was assessed using an XTT assay. Our statistical analysis revealed a significant dose- and time-dependent inhibitory effect of boric acid on TT cell viability. The IC50 of boric acid, the concentration at which cell viability is reduced by 50%, was determined to be 35 µM at the 48-h interval (*p* < 0.01). This threshold was statistically lower than the viability observed in the control group at the same time point (*p* < 0.01), and markedly different from the viability measures at lower concentrations of 10 µM and 25 µM at 24 h (*p* < 0.05 and *p* < 0.01, respectively), as well as at 72 h (*p* < 0.001 for both) (Fig. 1). In our analysis of the cytotoxic effects of boric acid on HThF cells, using a dose- and time-dependent framework, statistical outcomes indicated that cell viability remained consistently above 50% across all examined doses and time intervals, as confirmed by significant findings (*p* < 0.001). Specifically, even at the highest tested concentration of 500 µM, and extending up to 72 h of exposure, the viability threshold did not fall below 50%, highlighting the nuanced impact of boric acid on the resilience of HThF cells under the conditions studied (Fig. 2).

### mRNA Expression of Genes and miRNA by Real-Time PCR

Our analysis revealed that exposure to boric acid in TT cells resulted in a significant upregulation of genes related to apoptosis, including caspase 3, caspase 9, Bax, NOXA, and APAF-1 (respectively, *p* = 0.015, *p* = 0.046, *p* = 0.024, *p* = 0.022, *p* = 0.030). Conversely, there was a notable downregulation in the expression levels of Bcl-2 and Bcl-xl, which act as negative regulators of apoptosis (respectively, *p* = 0.038, *p* = 0.002). Additionally, there were statistically significant decreases in miRNA-21 and miRNA-224 (respectively, *p* = 0.020, *p* = 0.004) (Table 2).

**Table 2:** The mRNA expression changes of apoptosis genes, and miRNA mRNA expression in TT cancer cells treated with boric acid compared with the control group cells. Data were obtained by RT-PCR assay via ΔΔCt method in RT2 profile PCR array data analysis online program

Apoptosis-related genes		
*Gene names*	*Fold regulation*	*p-value*
Caspase-3	2.3613	**0.015**
Caspase-9	1.3332	**0.046**
BAX	4.2143	**0.024**
Bcl-2	− 2.1713	**0.038**
Bcl-xl	− 2.1624	**0.002**
NOXA	3.0652	**0.022**
APAF1	7.8504	**0.030**
Apoptosis-related miRNAs		
*miRNA names*	***Fold regulation***	***p-value***
miR-21-5p	− 4.3648	**0.020**
miR-224-5p	− 8.9801	**0.004**

*BAX* Bcl-2 associated X, *Bcl-2* B cell lymphoma 2, *Bcl-xl* B cell lymphoma-extra-large, *NOXA* phorbol-12-myristate-13-acetate-induced protein 1, *APAF1* apoptotic peptidase activating factor 1, *p* < 0.05 statistically significant

### Apoptotic Effects of Boric Acid in the TT Cell Line

**Fig. 3:**
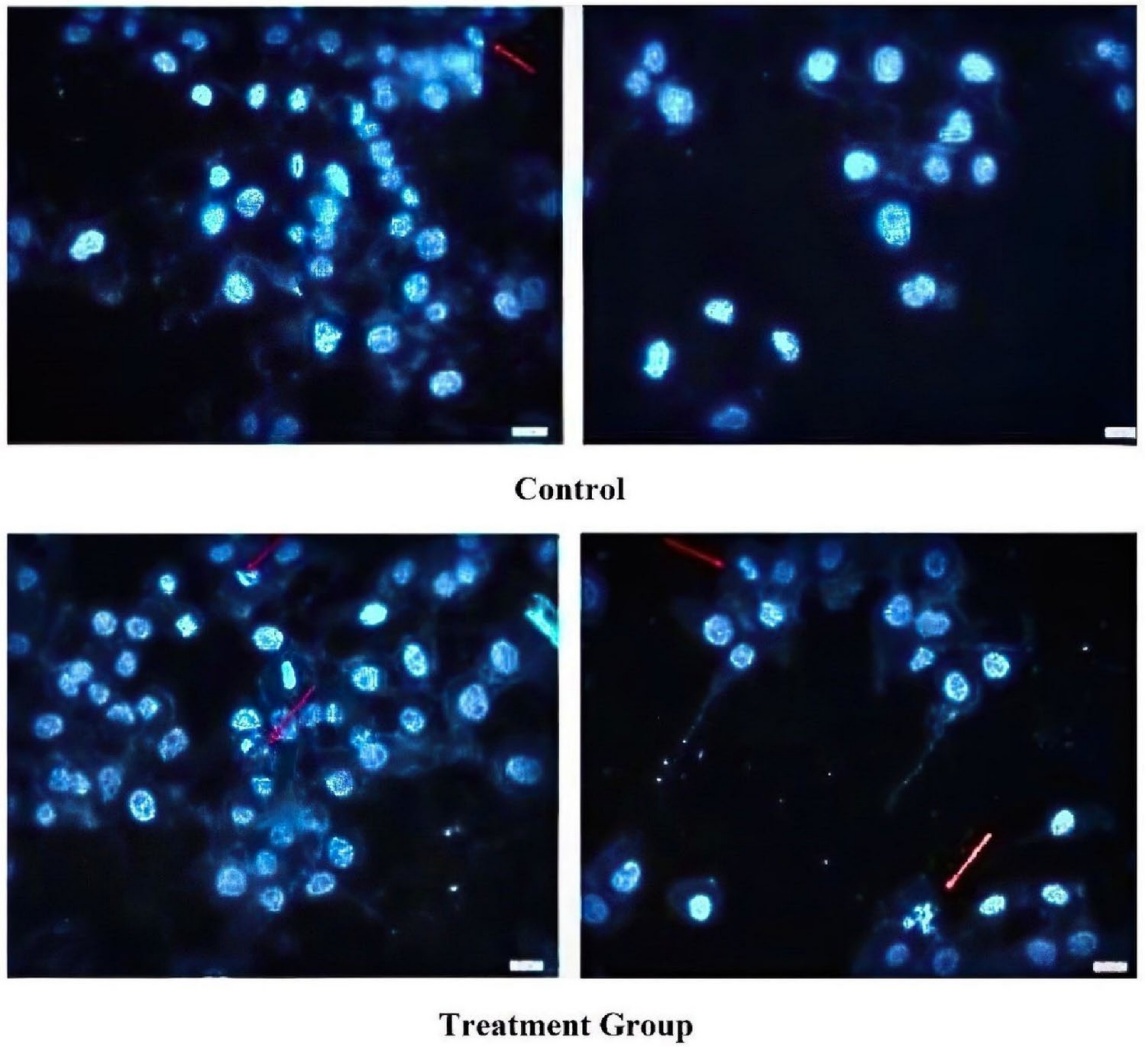
Fluorescence microscopy images at 20 × magnification of control and treatment group cells after Hoechst staining. The red arrows show the apoptotic cells (*p* = 0.026)

**Fig. 4:**
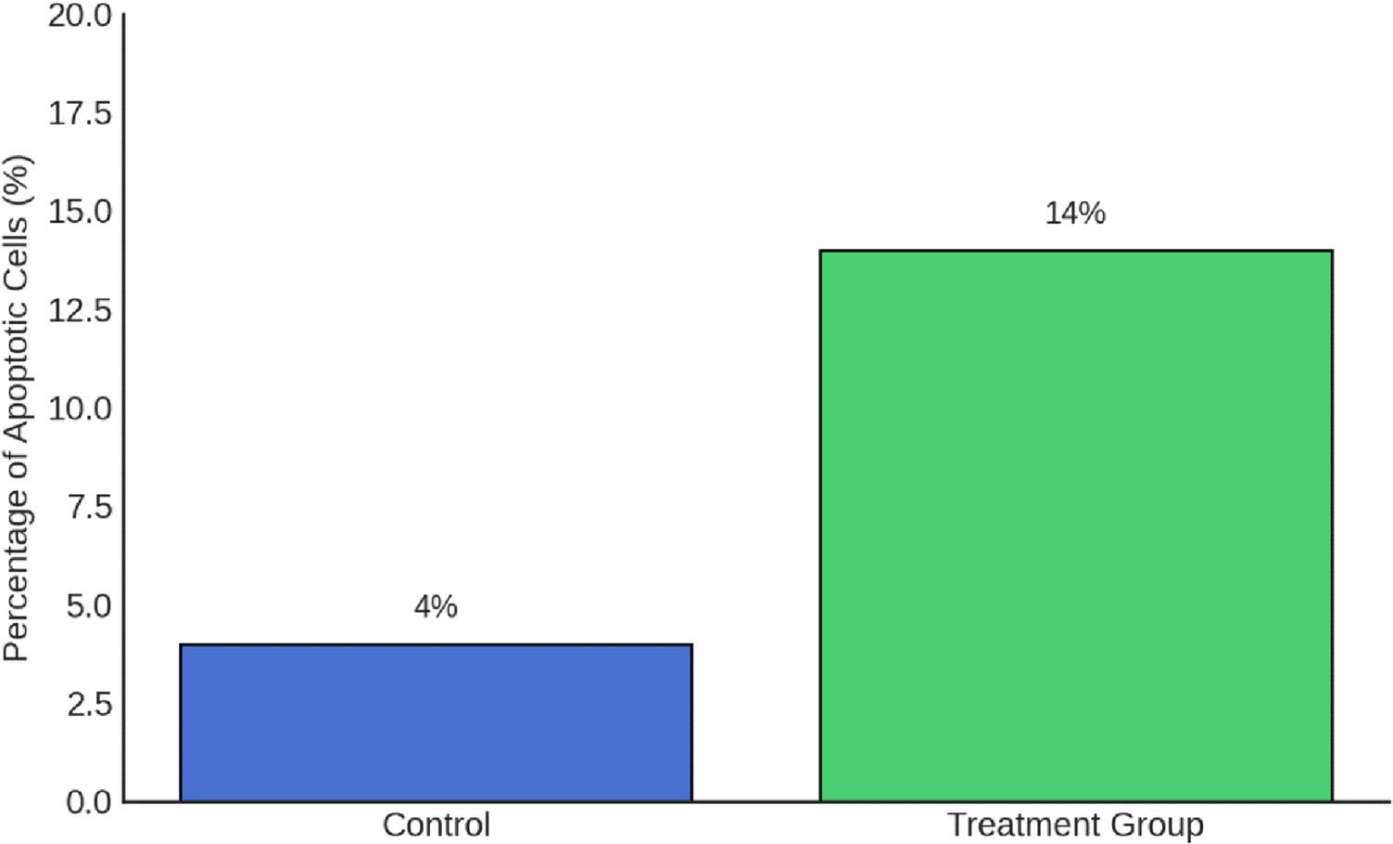
The percentage of apoptotic TT cells at 48 h was compared between the control and boric acid-treatment groups. The treatment group showed a significant increase in apoptosis (*p* = 0.026)

We compared the percentage of apoptotic cells between control and boric acid-treated TT cells. At the 48-h mark, following exposure to a concentration of 35 µM boric acid (identified as IC50 from XTT assays), a significant induction of apoptosis was observed. The control group exhibited a baseline apoptosis rate of 4%, whereas the treated group demonstrated a marked increase, with 14% of cells undergoing apoptosis (*p* = 0.026) (Figs. 3 and 4).

### Effects of Boric Acid on Colony Formation of the TT Cell Line

**Fig. 5:**
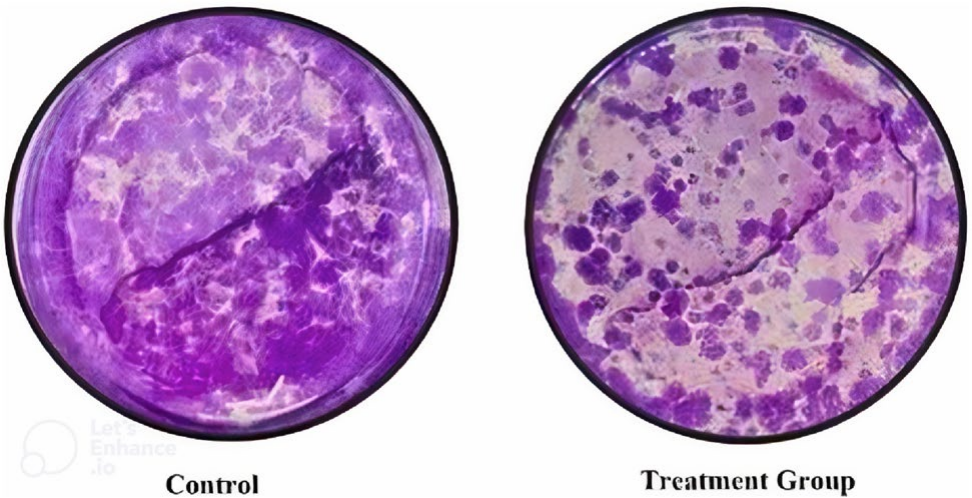
TT cell control and treatment group colony image (*p* < 0.001)

**Fig. 6:**
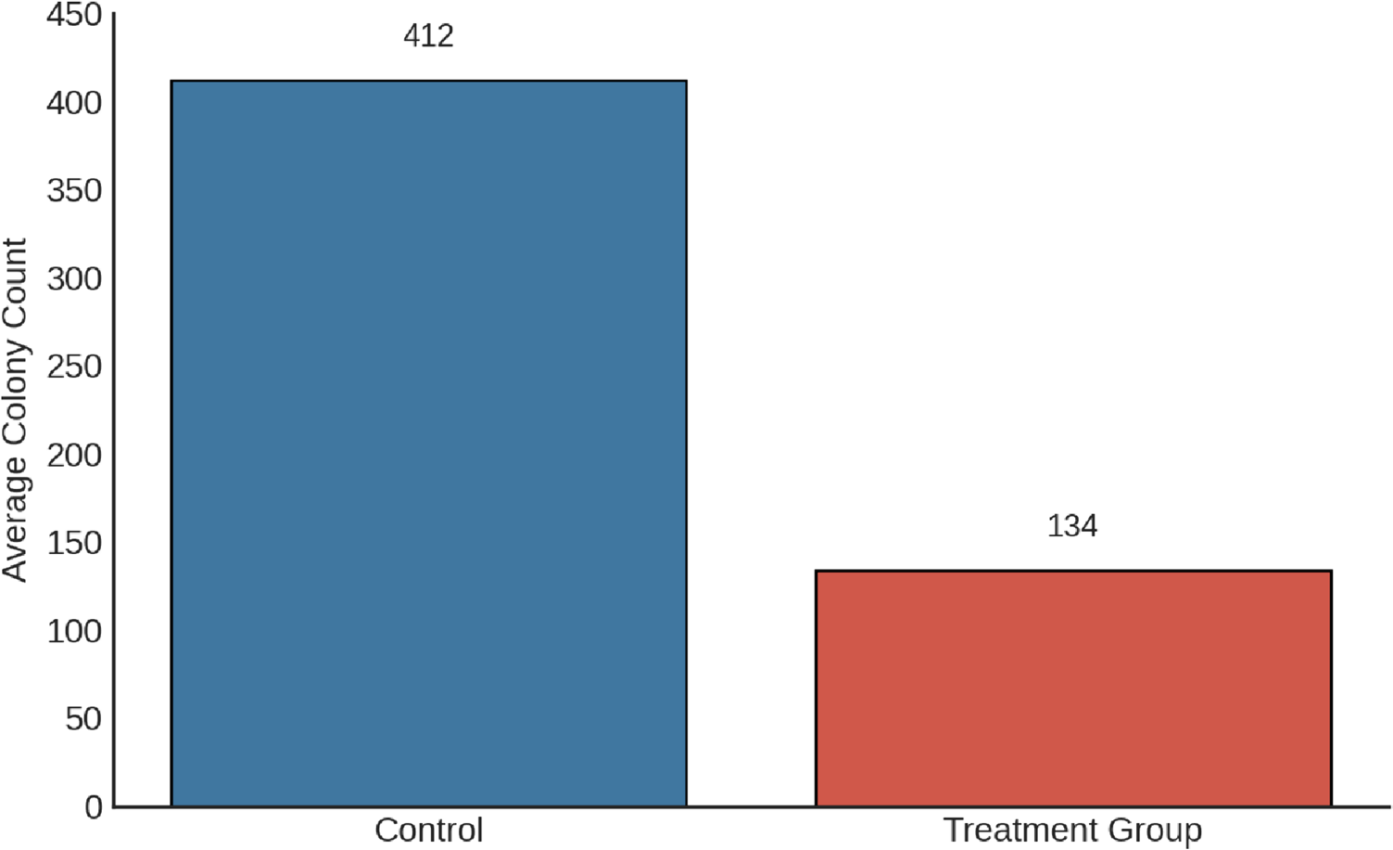
The average colony counts for control and treatment group, with the treatment group displaying a significant reduction in colony formation by 67.9% (*p* < 0.001)

A focused analysis was conducted at the 48-h time point using a singular, biologically relevant concentration of boric acid at 35 μM, which was identified as the IC50 dose from preliminary studies. The untreated control group had an average colony count of 412, serving as a baseline for comparison. In contrast, exposure to the 35 μM dose of boric acid resulted in a profound decrease in colony formation, with an average count of 134 colonies, representing a 67.9% reduction. The results indicate a significant decrease in colony formation of TT cells upon visual and quantitative assessment, demonstrating the inhibitory effect of boric acid on their proliferative capacity (*p* < 0.001) (Figs. 5 and 6).

## Discussion

The study examined the impact of boric acid on cell proliferation, colony formation, apoptosis, and the expression of genes and miRNAs related to the cell cycle and apoptosis. As there is a lack of research specifically investigating the effects of boric acid on MTC cells, our findings were compared to similar studies on other cancer cell lines available in the literature.

The cytotoxic impact of boric acid on TT cells was assessed, and an IC50 dose of 35 µM at 48 h was established. This specific dose served as the basis for subsequent experiments, which evaluated alterations in various cellular processes in comparison to control groups. In the conducted assays with XTT, the HThF cells showed a degree of cellular specificity in its anti-proliferative effects, with cell viability remaining above 60% for all concentrations. On the contrary, TT cells viability showed significant reduce between 35 and 500 µM dose of boric acid especially at 48 h and 72 h time intervals (*p* = 0.015). The cytotoxic effects of boric acid across various studies, including our own, consistently demonstrate a dose-dependent decrease in cell viability, although the specific impact varies by cell line and treatment duration. Similar to findings in DU-145, DMS-114, and SW480 cells identified a significant reduction in TT cell viability, particularly notable at medium to high concentrations over 48 to 72 h [9, 11, 12]. This pattern aligns with observations in other cancer cell lines, such as MDA-MB-231, where boric acid also showed a concentration-dependent cytotoxicity [13]. In contrast, studies like that by Wei Y et al. indicate that even low concentrations can be effective over extended periods, suggesting that both concentration and exposure time are critical factors in boric acid’s cytotoxic potential [16]. This variability underscores the importance of context in evaluating boric acid’s efficacy and highlights the need for tailored approaches depending on the specific cellular environment and treatment objectives.

Our investigation centered on the pivotal roles of *caspase-3*, caspase-8, and caspase-9 in mediating apoptosis within TT cells treated with boric acid. These caspases, crucial for both the extrinsic and intrinsic apoptotic pathways, serve as key executioners of apoptosis. *Caspase-3*’s role as a member of the cysteine-aspartic acid protease family is particularly notable for its central position in the cascade of apoptotic proteases, ultimately leading to cell death. [17, 18].

Boric acid’s influence on these caspases revealed intriguing patterns. Notably, our results indicated a significant upregulation in the expression of *caspase-3* and *caspase-9* upon boric acid treatment. This finding aligns with existing literature that underscores *caspase-3*’s activation as a hallmark of apoptosis induction. However, a unique aspect of our study is the concurrent increase in *caspase-9* expression, suggesting that boric acid may particularly emphasize the mitochondrial pathway of apoptosis. The increase in *caspase-3* activity observed in our study is consistent with the findings reported by Kahraman E. et al. and Hacioglu et al. This highlights boric acid as a significant activator of apoptosis by modulating *caspase-3*, a key executor of the apoptotic process [9, 19]. The findings suggest that boric acid consistently enhances *caspase-3* expression in various cancer models, including DU-145 and SW-480 cell lines, indicating a potentially universal *caspase-3* mediated apoptotic pathway induced by boric acid [11]. Furthermore, Cebeci et al. demonstrated an increase in mRNA levels of apoptotic genes, such as *caspase-3*, following boric acid treatment, which supports our findings [12]. Boric acid has the ability to trigger a broad-spectrum therapeutic response by modulating gene expression and enhancing the enzymatic activity of *caspase-3*.

The findings of our study, as well as other studies, suggest that boric acid is an effective inducer of apoptosis through a *caspase-3* mediated pathway. This indicates that it may have broad therapeutic potential across various types of cancer. The observed reduction in *caspase-9* activity indicates a complex interplay in boric acid’s apoptotic mechanism, which may affect both intrinsic and extrinsic pathways. Boric acid has the potential to provide targeted therapeutic strategies due to its distinct impact on caspase pathways. This highlights the importance of its complexity.

While many studies have reported on boric acid’s apoptotic effects through *caspase-3* activation, there are discrepancies in the results, with some research showing no significant impact on *caspase-3* levels [13]. These variations may be due to differences in cell types, experimental setups, or boric acid doses, indicating that the response to boric acid is context-dependent. This inconsistency calls for a cautious approach when interpreting the apoptotic potential of boric acid. The evidence, while compelling, is not unequivocal, and further research is needed to fully understand its effects [12, 15].

The *Bcl-2* gene family includes the anti-apoptotic proteins *Bcl-xl* and *Bcl-2*, which inhibit the release of cytochrome c, as well as *Bax*, a protein that promotes the release of pro-apoptotic factors from mitochondria [20]. Hinze et al. found strong expression of *Bcl-2* and moderate expression of *Bcl-xl* in MTC[21]. Our results demonstrate that boric acid treatment increases *Bax* mRNA expression and decreases *Bcl-2* and *Bcl-xl* mRNA levels in cells, which is consistent with previous studies [16, 22, 23]. Other studies have also reported increased Bax protein levels and decreased *Bcl-2* protein levels in response to boric acid treatment in different models, including rat colon tissues and DU-145 cells, indicating a consistent pro-apoptotic effect of boric acid across various systems [9, 24]. Overall, the literature and our findings consistently suggest that boric acid modulates apoptosis by altering the expression of key apoptotic regulators.

The expression of the pro-apoptotic *NOXA* gene, a member of the *Bcl-2* protein family, is regulated by the well-known tumor suppressor gene *p53* [25]. Previous research has shown that the RET proto-oncogene negatively regulates *NOXA* through the action of Transcription Factor 4 (*ATF4*) [26]. Furthermore, *APAF-1*, another factor related to apoptosis, forms a complex with cytochrome c that activates *caspase-9*, thereby inducing apoptosis [27]. In our study, a significant upregulation of both *NOXA* and *APAF-1* was observed in cells treated with boric acid. The upregulation of *caspase-3* and *caspase-9*, along with *NOXA* and *APAF-1*, supports a cohesive mechanism involving the intrinsic apoptosis pathway. *Caspase-9* is typically activated following *APAF-1*’s interaction with cytochrome c, leading to the activation of *caspase-3*, a key executioner in apoptosis. This cascade, potentially amplified by *NOXA*’s pro-apoptotic influence under *p53* regulation, underscores a detailed apoptotic response possibly unique to exposure to boric acid. This finding suggests a new apoptotic mechanism induced by boric acid, which has not been previously documented in the literature.

The complex biological role of boric acid is underscored by the differential impact it has on apoptosis across various cell lines, as observed in our study and others. Our investigation found that treating TT cells with an IC50 dose of 35 µM boric acid significantly increased apoptotic cell rates from 4% in the control group to 14% in the treated group, indicating a pro-apoptotic effect under these conditions, as revealed by a TUNEL assay (0=0.026). In contrast to Kobylewski et al.‘s observation, this finding suggests that the apoptotic effects of boric acid may be concentration-dependent, as lower concentrations (10 μM) reduced cell viability without triggering apoptosis in prostate cancer cells [28]. Additionally, studies with HepG2 cells and other cell lines indicate that boric acid can both inhibit proliferation and induce apoptosis, which differs from Scorei et al.’s findings where minimal apoptosis was noted [11–13, 16]. These differing outcomes may be attributed to variations in cell line susceptibility, experimental conditions, or boric acid concentrations. This highlights the necessity for further research to clarify the precise mechanisms by which boric acid affects cell viability and apoptosis. [13].

Pennelli and colleagues’ study on the *PDCD4*/*miR-21* pathway in MTC established a correlation between elevated *miR-21* expression and MTC progression. Higher levels of *miR-21* were linked to increased calcitonin levels, lymph node metastasis, and more advanced and resistant forms of the disease [29]. In another study by Chu et al., which involved 42 MTC cases, a significant overexpression of *miR-21* was observed in MTC cells compared to normal thyroid tissue. Moreover, the elimination of *miR-21* and *metastasis associated lung adenocarcinoma transcript 1 (MALAT-1)* from MTC cell cultures resulted in a significant reduction in both cell proliferation and invasion [30]. In Mian et al.‘s study, which included 34 sporadic MTC, six hereditary MTC, and two C cell hyperplasia cases, *miR-21* levels were found to be 4.2 times higher in affected tissue than in normal thyroid tissue [31]. Recent research has demonstrated that *miR-21* promotes cell proliferation by inhibiting tumor suppressor genes such as *PTEN*, *RECK*, *PDCD4*, and *TPM1* [32, 33]. In our study, a significant reduction in *miR-21* levels was observed in the treatment group compared to the control group (*p* = 0.02). The decrease in *miR-21* levels suggests interference with the *PDCD4/miR-21* pathway, which is known to influence medullary thyroid cancer progression. This reduction may suppress tumor-related processes such as cell proliferation and invasion by restoring function to tumor suppressor genes like *PTEN* and *PDCD4*, which are typically inhibited by *miR-21*. These findings suggest a potential therapeutic target for MTC by targeting *miR-21.*

Previous research on *miR-224* in MTC has shown that increased levels of *miR-224*are associated with the absence of lymph node metastasis, low-stage disease, and a favorable prognosis [34, 35]. However, studies on other types of cancer, such as breast, cervical, and lung cancer, have linked elevated *miR-224* expression to poor prognosis, increased cancer aggressiveness, and advanced disease stages [36]. In contrast, our study found a decrease in *miR-224* expression in TT cells treated with boric acid, which is unexpected given the generally positive association of *miR-224* with good prognosis and less aggressive disease in MTC (*p* = 0.004). This discrepancy raises questions about the role of *miR-224* in different cancer types and under varying treatment conditions. Although *miR-224* is typically associated with better outcomes in MTC, our study suggests that boric acid may influence other pathways or mechanisms that override its protective effects. Further research is needed to explore these dynamics and clarify the implications of *miR-224* modulation in cancer therapy.

Understanding the therapeutic potential of boric acid requires insight into its metabolic processing and absorption efficiency in the human body. Boric acid is absorbed through the gastrointestinal tract and skin, with its distribution primarily in blood, bones, and teeth. This bioavailability, influenced by factors like metabolic rate and health conditions, raises questions about the clinical feasibility of achieving the effective in vitro concentrations identified in our study. The IC50 dose of 35 µM for TT cells, while potentially achievable in the bloodstream, necessitates careful evaluation regarding safe and sustainable tissue concentrations. High doses have been associated with toxicity, highlighting the need for targeted delivery methods or localized treatments to maximize efficacy while minimizing systemic exposure. These considerations are crucial for translating in vitro findings into viable therapeutic strategies [37].

This study offers valuable insights into the effects of boric acid on medullary thyroid cancer cells, particularly highlighting its potential to influence apoptosis and gene expression. Despite these promising results, our findings are preliminary and should be interpreted with caution due to several limitations. The use of 2D cell culture assays may not fully represent the complex interactions and three-dimensional microenvironment of tumor cells *in vivo*. Additionally, our focus on a single cell line limits the generalizability of our results across different types of thyroid cancer or other cellular environments and the use of a single IC50 dose and a single time point in experiments limits our understanding of the dose-response relationship and temporal dynamics of boric acid’s effects. This approach may not capture the full range of cellular responses to varying concentrations and exposure times, which are crucial for establishing a comprehensive therapeutic index. To overcome these limitations, future studies should incorporate 3D culture systems, such as spheroids or organoids, which more accurately mimic the tumor microenvironment. Expanding the research to include a variety of thyroid cancer cell lines will help validate the broader applicability of our findings. Furthermore, investigating the differential effects of boric acid on cancerous versus normal cells will be crucial for understanding its therapeutic potential and safety profile. Comprehensive studies involving both in vitro and *in vivo* models are needed to elucidate the mechanisms by which boric acid modulates cell viability and apoptosis, ensuring a more robust evaluation of its clinical relevance in cancer therapy.

## Conclusion

The study shows that boric acid can inhibit cell proliferation and induce apoptosis in MTC through the modulation of miRNAs and apoptotic pathway elements. However, these results are preliminary, and further comprehensive in vitro and in vivo studies are necessary to confirm these effects and elucidate the underlying molecular mechanisms.

## Data Availability

The datasets generated during and/or analyzed during the current study are available from the corresponding author on reasonable request.
